# Dataset of mechanical properties from different types of wine stopper: Micro-agglomerated, natural cork and synthetic

**DOI:** 10.1016/j.dib.2018.11.051

**Published:** 2018-11-16

**Authors:** Mariola Sánchez-González, David Pérez-Terrazas

**Affiliations:** INIA-CIFOR, Ctra. de La Coruña, km 7,5, 28040 Madrid, Spain

## Abstract

The data in this paper are related to the research article entitled “Assessing the percentage of cork that a stopper should have from a mechanical perspective” (González and Terrazas, 2018). This data article contains data on the mechanical properties of different types of wine stoppers: 18 types of micro-agglomerated stoppers, three types of natural stoppers, and one type of co-extruded synthetic closure. Mechanical properties were evaluated with different analysis: Compression test for the maximum radial compression force, the young’s moduli and the diameter recovery, relaxation test for the relaxation force and the extraction test for the displacement force.

**Specifications table**

**Table:** 

Subject area	*Agricultural and Biological Sciences*
More specific subject area	*Food science*
Type of data	*Tables and graphs*
How data was acquired	*Zwick universal testing machine with a 20,000 N load cell, a device developed in the INIA-CIFOR Cork Laboratory with a 150 kg load cell and a Mitutoyo ID-F150 digital vernier callipers*
Data format	*Analyzed*
Experimental factors	*Cork percentage from 0% (synthetic closures) to 100% (natural cork stoppers) and micro-agglomerated cork stoppers with different percentages of cork in their formulation*
Experimental features	*Different mechanical tests were carried out simulating the bottling process, the beginning of the sealing period and the extraction process*
Data source location	*Spain*
Data accessibility	*Data is with this article*
Related research article	*M. Sánchez González and D. Perez Terrazas, Assessing the percentage of cork that a stopper should have from a mechanical perspective, Food Packaging and Shelf Life (2018) In Press.*[1]

**Value of the data**

• Data show the mechanical properties of stoppers, which are valuable for assessing sealing performance of wine stoppers, through different tests that simulate the bottling process, the beginning of the sealing period and the extraction process.
• These data can be used to compare the mechanical performance of different types of wine stoppers micro-agglomerated, natural and synthetic.
• These data are suitable to support decision-making of producers and consumers in choosing suitable wine stoppers.

## 1 Data

In the data, Table 1, Table 2 show the different formulation of the micro-agglomerated cork stopper groups tested and the dimensions and density for each cork stopper type, respectively.

**Table 1:** Formulation of the micro-agglomerated cork stopper groups tested.

Sample code	Cork (%)	Binder (%)	Stopper density (kg·m^−3^)
1A	90	10	230
1B			290
1C			350
1D	80	20	230
1E			290
1F			350
1G	70	30	230
1H			290
1I			350
1J	60	40	230
1K			290
1L			350
1M	50	50	230
1N			290
1O			350
1P	40	60	230
1Q			290
1R			350

**Table 2:** Characterization of dimensions and density for each cork stopper type (min: minimum; max: maximum; std: standard deviation).

Stopper type	Longitude (cm)				Diameter (cm)				Stopper density (kg·m^−3^)			
	Media	Min	Max	Std	Media	Min	Max	Std	Media	Min	Max	Std
1A	44.07	43.98	44.17	0.05	24.20	24.10	24.28	0.04	230.73	224.19	238.64	4.33
1B	44.09	44.03	44.27	0.04	24.25	24.20	24.34	0.03	286.23	278.65	293.14	3.55
1C	44.01	43.88	44.23	0.08	24.26	24.18	24.34	0.05	339.63	328.44	351.52	4.70
1D	44.12	44.04	44.18	0.04	24.24	24.17	24.29	0.03	230.13	223.12	238.96	3.95
1E	44.12	44.07	44.17	0.02	24.24	24.19	24.28	0.03	286.95	279.43	294.81	4.78
1F	44.00	43.94	44.05	0.03	24.17	24.12	24.21	0.02	354.15	345.06	360.95	4.23
1G	43.98	43.91	44.05	0.04	24.15	24.05	24.23	0.05	233.33	225.36	239.92	3.49
1H	43.94	43.90	44.14	0.05	24.14	24.10	24.33	0.04	290.29	284.57	297.98	4.13
1I	43.74	43.35	44.11	0.23	24.22	24.12	24.29	0.05	353.59	344.79	365.60	5.51
1J	44.12	44.04	44.19	0.03	24.13	23.70	24.27	0.11	235.16	229.72	243.05	2.76
1K	44.06	43.86	44.17	0.05	24.18	24.03	24.26	0.04	292.98	280.21	299.22	3.80
1L	43.52	43.27	43.70	0.11	24.08	23.78	24.17	0.06	360.47	346.70	373.78	5.51
1M	43.51	43.34	43.73	0.10	24.18	24.12	24.23	0.03	229.91	225.36	232.41	1.48
1N	44.06	43.93	44.18	0.06	24.10	23.95	24.25	0.07	294.15	285.46	300.59	3.44
1O	43.67	43.07	43.82	0.12	24.07	23.84	24.14	0.05	362.39	351.79	368.59	4.23
1P	43.76	43.70	43.83	0.03	24.18	24.14	24.20	0.01	232.34	227.32	239.78	3.24
1Q	44.01	43.81	44.15	0.06	23.92	23.46	24.06	0.12	297.18	289.40	311.48	5.02
1R	43.80	43.68	43.92	0.06	24.03	23.79	24.11	0.06	359.76	352.32	368.05	4.78
NH	44.38	43.71	45.06	0.36	23.74	23.42	23.96	0.15	167.34	152.21	194.25	10.35
NR	44.30	43.79	44.93	0.37	23.68	23.30	24.08	0.17	170.12	151.68	188.52	11.37
NW	44.28	43.75	44.94	0.33	23.70	23.27	23.99	0.18	172.89	147.81	199.99	12.99
SC	42.67	42.42	43.27	0.16	23.43	23.24	23.77	0.12	301.88	293.72	307.37	2.51

**Fig. 1:**
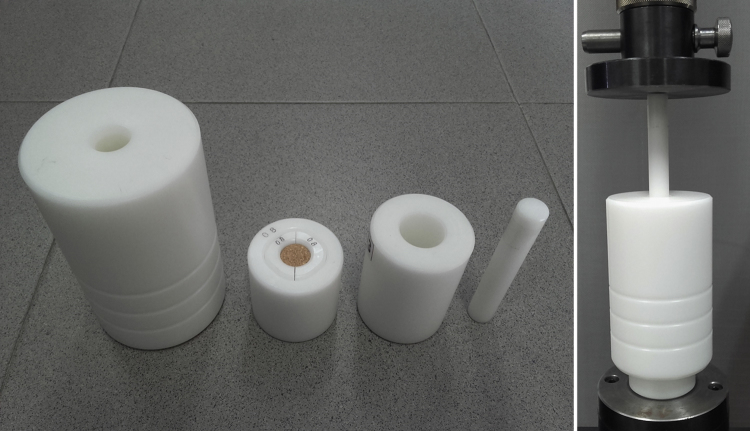
Device developed in the INIA-CIFOR Cork Laboratory for measuring the displacement force. From left to right: external cylinder; bottleneck simulation tube; receptor cylinder; plunger; and the whole device positioned on the universal testing machine.

**Fig. 2:**
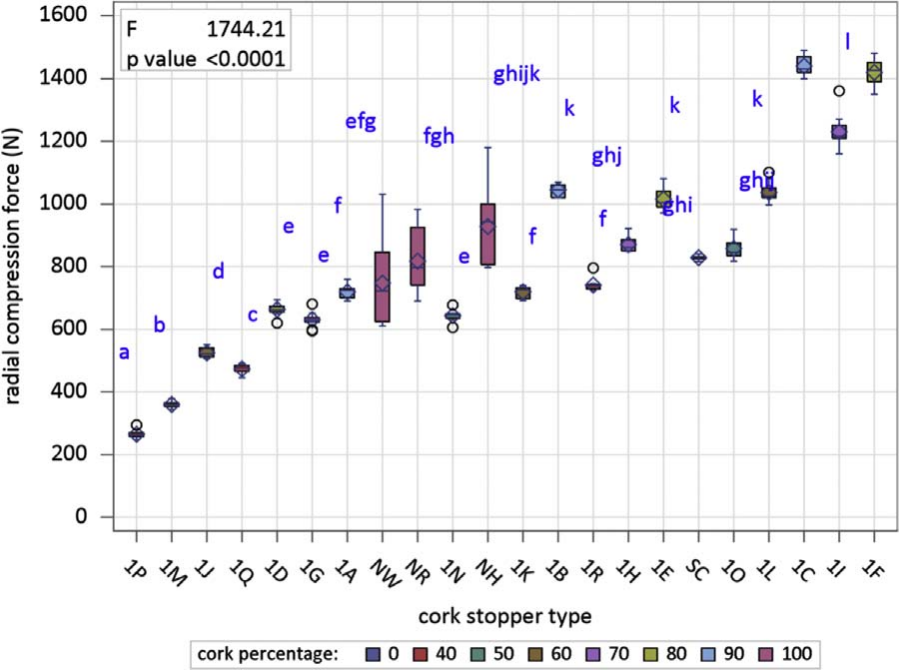
Boxplots of maximum compression force distributions per stopper type. Boxplot notches indicate a 95% confidence interval on the median.

**Fig. 3:**
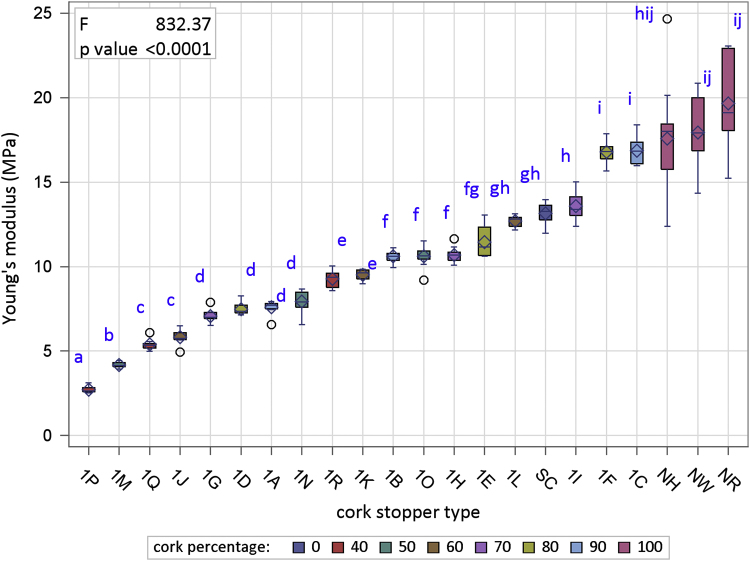
Boxplots of Young’s modulus distributions per stopper type. Boxplot notches indicate a 95% confidence interval on the median.

**Fig. 4:**
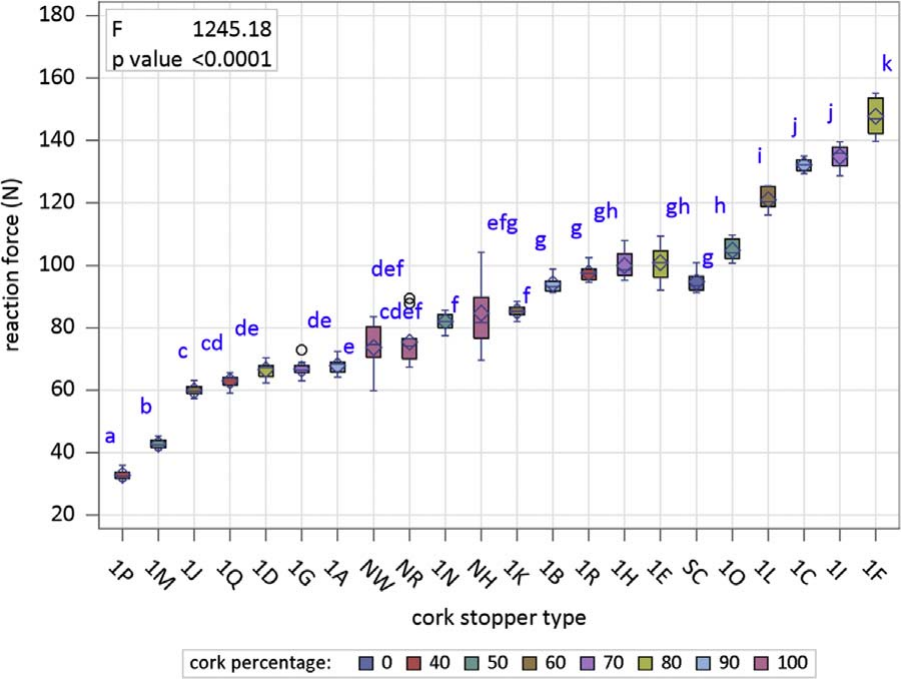
Boxplots of reaction force distributions for stopper type. Boxplot notches indicate a 95% confidence interval on the median.

**Fig. 5:**
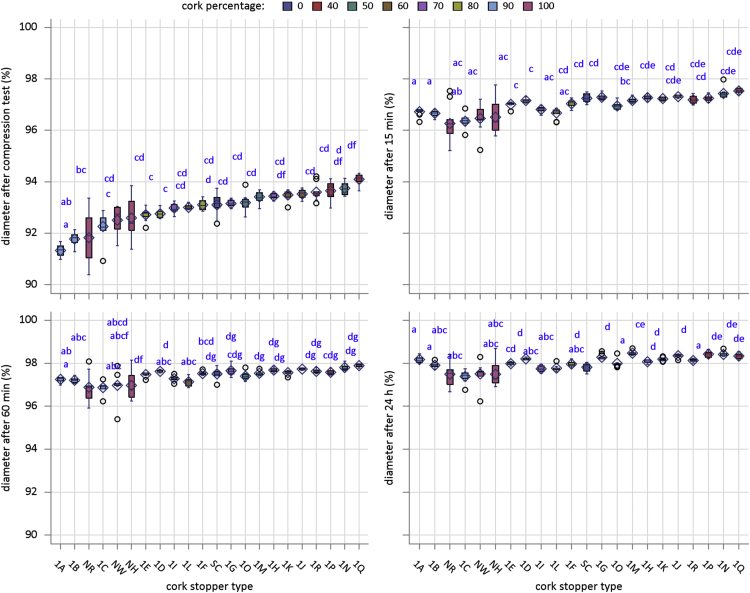
Boxplots of diameter recovery distributions per stopper type. Boxplot notches indicate a 95% confidence interval on the median.

**Fig. 6:**
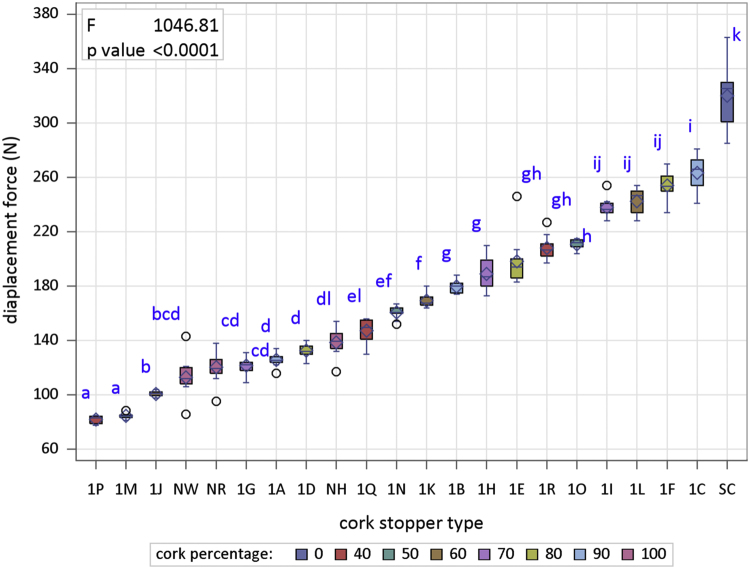
Boxplots of displacement force distributions per stopper type. Boxplot notches indicate a 95% confidence interval on the median.

Fig. 1 is the device developed in the INIA-CIFOR Cork Laboratory for measuring the displacement force. Fig. 2, Fig. 3 show the maximum compression force distributions and the Young’s modulus distributions per stopper type, respectively. Fig. 4 shows the reaction force distributions for each stopper type. Fig. 5 shows the diameter recovery after compression test and after 15, 60, and 1,440 min. for each stopper type. Fig. 6 shows the displacement force distributions per stopper type. In Fig. 2, Fig. 3, Fig. 4, Fig. 5, Fig. 6 the boxplot notches indicate a 95% confidence interval on the median.

## 2 Experimental design, materials, and methods

This article shows the mechanical properties from 22 types of cylindrical wine stoppers, 18 types of micro-agglomerated stoppers, three types of natural stoppers, and one type of co-extruded synthetic closure. Micro-agglomerated stoppers differing cork mass percentages (90, 80, 70, 60, 50, and 40%) and the densities 230, 290 y 350 kg·m^−3^ (6 formulations×3 densities [sample code: 1A-1R; Table 1]). Natural cork stoppers differing the external visual (sample code: NH, NR, NW). The sample code for synthetic closures is SC.

Once acclimatized (20 °C and 65% of relative humidity), stoppers were weighed and measured (Table 2) using Mitutoyo ID-F150 digital vernier callipers. Stoppers density was calculated as already reported in González-Hernández [2].

### 2.1 Uniaxial compression test

The maximum radial compression force (compress each stopper to 33% of the initial diameter) was measured using a Zwick universal testing machine (Zwick GmbH & Co.) with a 20,000 N load cell. A stress-strain curve was drawn from the data recorded and Young׳s modulus was calculated for each of the stoppers tested as the slope of the linear elastic portion of the stress-strain curve between 1% and 2% of the strain [3].

Diameter recovery was measured immediately after the compression test and again after 15 min, 1 h and 24 h after the test using a Mitutoyo ID-F150 digital vernier callipers.

### 2.2 Relaxation test

As already reported in Sánchez González [1], the relaxation force [2] was measured with a device developed in the INIA-CIFOR Cork Laboratory [4].

### 2.3 Extraction test

The displacement force [1] is the maximum force required to extract the stopper and is a proxy of the extraction force. This force was measured by a device developed in the INIA-CIFOR Cork Laboratory (Fig. 1) used in the Zwick universal testing machine as already reported in Sánchez González [1]. All stoppers used in this test were previously surface treated with an aqueous emulsion comprising silicones and waxes.

### 2.4 Statistical analysis

All tests were carried out using the SAS software version 9.4 as already reported in Sánchez González [1].
